# Community Health Worker Diabetes Prevention Awareness Training in an Immersive Virtual World Environment: Mixed Methods Pilot Study

**DOI:** 10.2196/64051

**Published:** 2025-06-10

**Authors:** Laurie Ruggiero, Lauretta Quinn, Amparo Castillo, Colleen Monahan, Leticia Boughton Price, Wandy Hernandez

**Affiliations:** 1 Health Behavior and Nutrition Sciences, College Of Health Sciences, University of Delaware, Rm 315, 100 Discovery Blvd, Newark, DE, 19716, United States, 1 302-831-8506; 2 Biobehavioral Nursing Science, College of Nursing, University of Illinois at Chicago, Chicago, IL, United States; 3 Community Health Sciences, School of Public Health, University of Illinois at Chicago, Chicago, IL, United States; 4 School of Public Health, University of Illinois at Chicago, Chicago, IL, United States; 5 Illinois Community Health Workers Association, Chicago, IL, United States

**Keywords:** diabetes prevention, virtual world, community health workers, African American, training, community health worker, pilot study, obesity, community awareness, remote training, mixed-methods, acceptability, feasibility

## Abstract

**Background:**

The burden of diabetes and obesity are greater for some racial-ethnic minority groups in the United States, including non-Hispanic blacks, underscoring the importance of raising community awareness of diabetes prevention. Community health workers (CHWs) play a critical role in extending our reach into communities to raise awareness of diabetes prevention. Systematic training and support are central to their work. Remote approaches have been helpful in delivering training to overcome common participation barriers. One remote approach, immersive 3D virtual worlds (VW) offer a unique approach to providing remote training incorporating engaging interactive contextual learning opportunities.

**Objective:**

This study aimed to implement and evaluate an internet-based 3D VW model to remotely deliver an adapted CHW training program on diabetes prevention awareness for racial-ethnic minority communities.

**Methods:**

A sequential mixed methods design, including a pre-post pilot and explanatory phase, examined the feasibility, acceptability, and impact of the VW training. Female CHWs who self-identified as African American or Black or African Ancestry, between 21‐65 years of age, fluent in English, and with risk factors for diabetes were recruited. CHW input was gathered to adapt a Centers for Disease Control and Prevention’s CHW diabetes prevention awareness training and the VW environment for this study. The final adapted training was standardized for delivery over 10 weeks. Quantitative and qualitative data were collected to examine acceptability, feasibility, and impact of the training model. Primary quantitative pre-post outcomes included training content knowledge and confidence; and secondary behavioral outcomes included motivation for lifestyle change and eating habits. Focus group feedback was collected on acceptability and feasibility during the explanatory phase. Quantitative descriptive and qualitative thematic analysis approaches were used to examine the acceptability, feasibility, and impact of the VW training model.

**Results:**

A total of 26 CHWs initiated the study and 22 completed the postassessment. The majority of participants reported that their expectations were met across all sessions and content topics. Participants generally reported satisfaction with the information provided (20/22, 91% rated very good-excellent) and high levels of interactivity in the training (17/22, 77% rated very good-excellent). Results of the posttraining acceptability and feasibility quantitative survey and qualitative feedback were generally positive. Mean pre-post values improved across all quantitative outcomes for the VW training group (eg, 92% [11/12] improved in knowledge; 62% [8/13]‐77% [10/13] improved across eating habits measures). Explanatory focus group findings were generally positive, highlighting satisfaction with the overall training, its interactivity, and content. The main constructive feedback was related to providing more training and support in using the avatar.

**Conclusions:**

Findings on the acceptability, feasibility, and preliminary impact of the VW training model are promising and support continued use, development, and research on this approach.

## Introduction

### Obesity and Diabetes Risk

The burden of diabetes and obesity are greater for racial-ethnic minority groups in the United States, including non-Hispanic Blacks [1,2]. Estimates (2019‐2021) of the prevalence of diagnosed diabetes in the United States indicate that 12.5% of Black non-Hispanic adults (>18 years) have diabetes compared with 8.5% of white non-Hispanic adults [3]. Obesity is a major risk factor for the development of type 2 diabetes mellitus (T2DM). Estimates from the National Health and Nutrition Examination Survey (2017‐2020) indicated that Black non-Hispanic adults (>20 years) had the highest prevalence rates of obesity (49.9%) compared with non-Hispanic Whites (41.4%), non-Hispanic Asian (16.1%), and Hispanic individuals (45.5%) [4]. The greater prevalence of obesity and diabetes underscores the importance of raising awareness of diabetes and its risk in this racial-ethnic group and connecting individuals with available evidence-based programs, such as the National Diabetes Prevention Program [5].

### Community Health Workers Extend Our Reach in Underserved Communities

The Community health worker (CHW) Section of the American Public Health Association describes CHWs in the following way: “frontline public health worker who is a trusted member of and/or has an unusually close understanding of the community served. This trusting relationship enables the worker to serve as a liaison or link or intermediary between health or social services and the community to facilitate access to services and improve the quality and cultural competence of service delivery. A CHW also builds individual and community capacity by increasing health knowledge and self-sufficiency through a range of activities such as outreach, community education, informal counseling, social support and advocacy” [6]. CHWs generally share characteristics of the community they serve, such as language, race-ethnicity, and lived experience. Evidence has been accumulating on the role and impact of CHWs involvement in both supporting people with diabetes [7-10] and promoting diabetes prevention [11-13]. Equipping and supporting CHWs in raising community awareness about diabetes risk factors and prevention in higher risk and underserved communities can support the public health effort to improve health equity.

### Training and Empowering CHWs to Support Diabetes Prevention Efforts

Systematic training and support are central to the work of CHWs [14]. Studies have described different training approaches, and some have examined the link between training and outcomes [15]. Research comparing training approaches is limited and a recent systematic review underscored the lack of research specifically examining CHW training approaches and their impact with African American communities [16].

Remote approaches have been helpful in delivering training that maximizes the reach and may help overcome common barriers to participation (eg, transportation and childcare) and novel unanticipated participation barriers, such as experienced during the COVID-19 pandemic. The value of remote approaches to working and providing training was clearly recognized during the COVID-19 pandemic [17]. The use of video conferencing has become a regular tool to reach CHWs and help remove participation barriers to training. Another remote approach, immersive 3D virtual worlds (ie, using avatars), offers a unique opportunity to provide remote training, while also offering additional engaging interactive contextual training opportunities.

### Leveraging the Virtual World to Provide Remote Training

Internet-based immersive 3D virtual worlds (eg, Second Life) offer the opportunity to reach populations with standardized and tailored training that can overcome common barriers to participation and allow for engaging, interactive, and contextual or experiential educational opportunities. Individuals can synchronously interact with each other (using voice or text chat) and the learning environment through avatars. A virtual world (VW) model has many potential advantages in offering trainings for CHWs: (1) scalable to disseminate to diverse populations remotely; (2) supports internal validity (eg, standardized delivery); (3) removes common barriers to participation (eg, transportation time or expense, child care); (4) can be tailored (eg, virtual environment, activities, content) for diverse groups; (5) provides engaging interactive contextual or experiential learning opportunities to build knowledge, skill, confidence, and empathy; and (6) allows for social interaction and peer-to-peer learning; and participation does not require special equipment (other than a computer). Research has demonstrated the use of Second Life to deliver health education in general [18] and a few published studies have shown promise of its use to help people make healthy lifestyle changes for weight loss or to provide diabetes self-care education [19-23]. To our knowledge, no published research has examined the use of this immersive 3D VW delivery model for implementing a CHW training, in general, or specifically to raise awareness about diabetes prevention to help extend efforts in underserved at-risk racial-ethnic minority communities.

### Objective and Aims

Our long-term objective is to identify effective strategies to promote healthy lifestyle change to reduce obesity and diabetes risk in underserved populations and communities. The overall aims of this study were to adapt, implement, and evaluate a VW model to remotely deliver an adapted best-practice CHW training (based on Center for Disease Control and Prevention’s “Road to Health Toolkit”) [Road to Health; RTH] to support CHWs efforts to raise awareness about diabetes prevention in racial-ethnic minority communities.

This study was conducted before and during the COVID-19 pandemic (details described later). The original prepandemic aims were to conduct a randomized implementation pilot study to examine change in primary outcomes (0-12 wk) after the in-person (RTH) or VW delivery (RTH-VW) of the training program; and examine feasibility and acceptability of the VW delivery of the RTH training. The original hypotheses were: the 2 training approaches would result in similar preliminary outcomes (ie, equivalency); and the VW delivery would be feasible and acceptable to CHWs. We were unable to complete the randomized controlled trial as planned because of COVID-19 pandemic restrictions, therefore, all subsequently enrolled participants were assigned to the RTH-VW. This paper describes all available study participants (RTH and RTH-VW) and focuses on the RTH-VW group.

## Methods

### Design

A randomized 2-group (RTH and RTH-VW) repeated measures design (baseline and posttraining) was initially implemented (prepandemic). COVID-19 pandemic-related study delays (ie, 9 mo) and challenges (eg, delayed groups; inability to hold in person meetings) led to the modification of the experimental design to a quasi-experimental single-group pre-post design (RTH-VW only).

A sequential mixed methods research design [24] incorporating both quantitative and qualitative approaches was used. This paper describes a pre-post pilot phase examining acceptability, feasibility, and the preliminary impact of the training, followed by an explanatory phase clarifying and expanding upon the findings of the pilot. The primary quantitative variables included primary training-related outcomes (ie, knowledge and confidence). An additional objective of the study was to include CHWs who experience risk factors similar to the population they serve and to offer the opportunity to personally implement the lifestyle changes learned to help facilitate experiential learning and empathy for the challenges that may be experienced by others making similar lifestyle changes. Therefore, we also examined secondary behavior change outcomes.

### Training Description

#### Adapted CHW Diabetes Prevention Training

We chose the Center for Disease Control and Prevention’s (CDC) Division of Diabetes Translation’s (DDT) RTH Toolkit [25] as the CHW training program. The 2008 version was used since it was the only version available at the start of the study (updated version now available). It was chosen because it was developed with CHW collaboration and focus group testing; incorporates storytelling, literacy tailoring and cultural tailoring for African American communities; and includes a comprehensive set of resources to support standard implementation. The overall RTH training goals are to raise awareness of diabetes, its risk factors, and prevention activities (making healthy food choices and physical activity) to reduce weight in those overweight or obese to help lower risk of T2DM. The RTH Toolkit content and resources adapted for this study served as the core CHW training.

**Figure 1.**
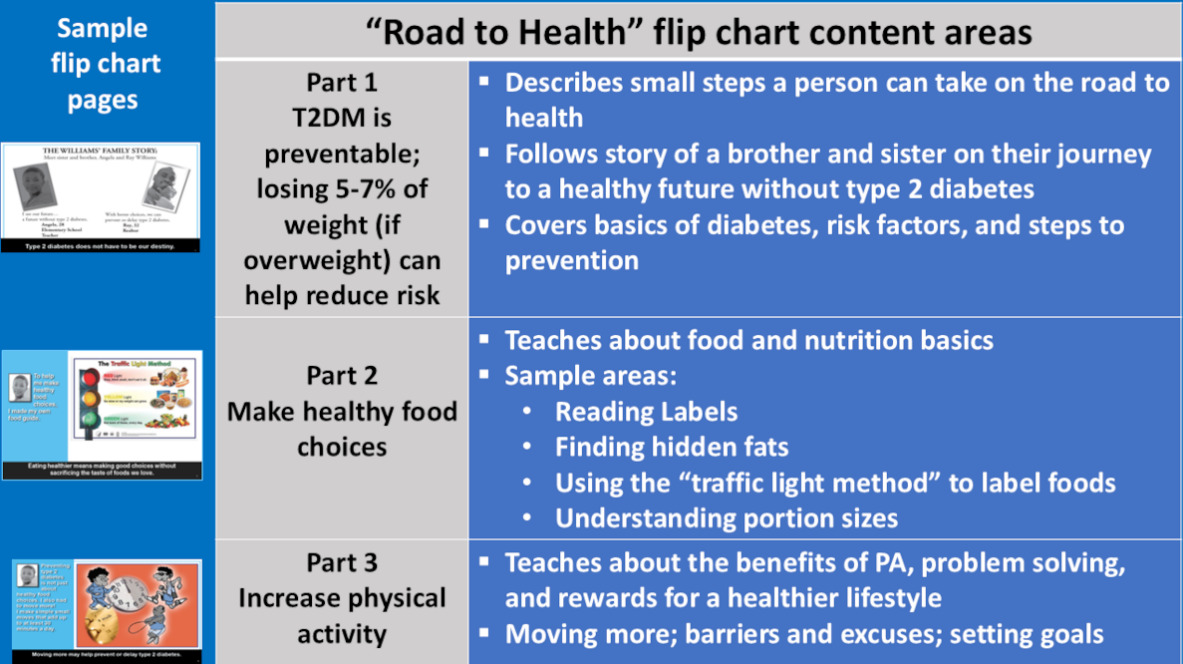
Diabetes prevention awareness primary content areas and sample topics from the “Road to Health Toolkit” adapted for the CHW training. Note: This study used the 2008 CDC “Road to Health Toolkit” and graphics are from that version; an updated version is available. T2DM: type 2 diabetes mellitus.

The 3 main diabetes prevention messages of the RTH toolkit were used and can be seen in Figure 1 (based on 2008 version). Resources to support CHW participation and facilitate diabetes prevention community awareness efforts were included: (1) original resources from the RTH Toolkit [26] (eg, training and resource guides, posters, eating and activity tracking tools, and flip chart) and (2) supplemental content-related resources (eg, American Diabetes Association and CDC prediabetes risk test [27], US Department of Agriculture MyPlate resources [28], and US Food and Drug Administration nutrition facts label information [29].

Based on an intensive community-engaged development phase incorporating CHW input over several months to gather feedback on iterative versions of adapted training and VW environment, the final training was adapted and standardized for delivery over 10 weeks with an orientation or overview session, 8 content sessions delivered biweekly (2 topics or session) and final summary session. The 10-week duration of the training was designed to allow CHWs the opportunity to try the strategies learned in their own lives, if desired, to gain personal experience in lifestyle change.

#### VW Description

The VW environment used was Second Life, a computer-based simulated 3D environment, intended for users to inhabit and interact via an avatar (VW representation of the user). Users are identified by their assigned avatar name and can communicate with other users (through avatars) in real time using voice and text chat tools. Real life anonymity is possible unless the individual chooses to share their real-world identity. Based on CHW input in the development phase of this project, we adapted the VW environment to support delivery of the RTH training. The Second Life VW created for the study island was private. Only study participants and research staff (as avatars) had access and study avatars could not travel to other spaces in Second Life.

**Figure 2.**
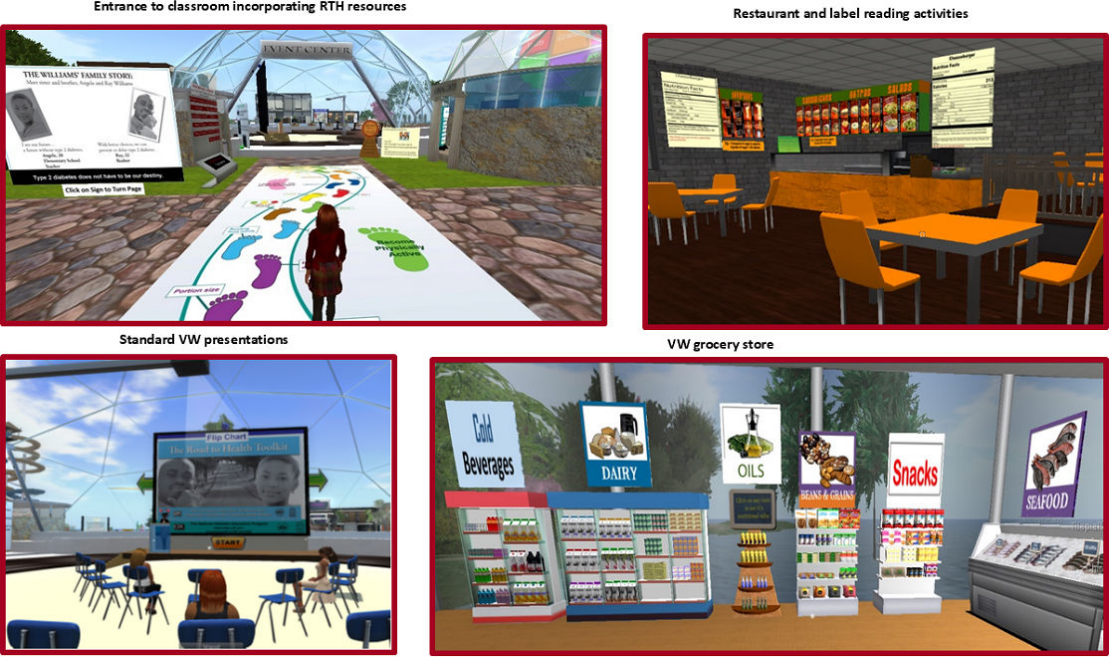
Examples of the virtual world environment and activities. This study used the 2008 CDC “Road to Health Toolkit” and graphics are from that version; an updated version is available. RTH: Road to Health Toolkit; VW: virtual worlds.

The final adapted environment included synchronous educational sessions in the VW classroom or in other VW locations (eg, fast food restaurant), immersive experiential activities (eg, comparing food labels in grocery store), access to educational resources, educational messages posted throughout the virtual environment (eg, MyPlate messages in grocery store), peer interactions, and knowledge self-checks opportunities. Example scenes from the VW environment are shown in Figure 2.

#### Study Population and Recruitment.

The eligibility criteria included: female, self-identify as African American or Black or African Ancestry; adult (21‐65 y old); self-report as overweight or obese based on BMI; currently inactive, defined as not participating in regular moderate physical activity (150 min/wk), self-identify as a CHW, fluent in English, and regular access to a computer and the internet. The CHWs were purposefully recruited to share characteristics (eg, diabetes risk factors) to facilitate an understanding of the lived experience of the community members they are working to reach with messages about diabetes prevention awareness. This approach offered an opportunity for participants to implement the knowledge and lessons learned in their own lives to make lifestyle changes and gain understanding of and empathy for the process and challenges of making healthy lifestyle changes. Exclusion criteria included: (1) household member already participating in study, (2) plans to travel or move from the city during the study period, and (3) self-reported inability to participate in regular moderate physical activity.

CHW participants were recruited in collaboration with the Illinois Community Health Worker Association (ILCHWA). Project recruitment information was shared with potential participants through researcher-led informational sessions and distribution of study flyers at CHW professional meetings in the Chicago-area, and using various communication strategies (eg, email and social networking approaches) implemented by the ILCHWA to share the study flyer. Similar recruitment approaches also occurred through research team connections and communications with various CHW community and academic groups, health systems employing CHWs, and CHW training programs in the Chicago area.

### Ethical Considerations

Interested individuals were instructed to call a study phone number to get more information about the study and to determine their eligibility for study participation. University of Illinois at Chicago (UIC) research team members conducted phone eligibility screening. Eligible and interested individuals attended an in-person meeting at UIC (prepandemic) or via teleconferencing (during pandemic) to complete the study orientation, written paper consent (eg, completed digitally using REDCap [30,31] during pandemic). Study staff fully informed individuals of the study (eg, procedures, incentives, and assessments), addressed questions, and obtained consent. Participants received US $25 for completing the first in-person assessment and US $100 for completing the postintervention in-person assessment. An additional US $25 was provided for focus group (described below) participation. To maximize retention at assessments, announcements were made during program sessions and follow-up prompts were conducted. Efforts were made to minimize barriers to participation (eg, parking reimbursement) for in-person meetings. This study involved human participants; the study protocol was reviewed and approved by the University of Delaware (#1106606‐20) and University of Illinois (IRB 2018‐0986) institutional review boards.

### Implementation

Following the baseline assessment and (pre-COVID-19 pandemic), CHWs were randomized to either the in-person or VW CHW training program. After assessment and randomization, participants took part in the CHW training either virtually (RTH-VW) or in person (RTH). For both the RTH and RTH-VW, the training sessions were delivered in small cohorts (ie, up to 11) and led by the same facilitator (ie, Registered Dietitian) who had extensive experience in delivering group health education programs with diverse populations, both in person and in the 3D VW environment. Before COVID-19 restrictions, the facilitator and CHWs participated as avatars in the VW for the RTH-VW groups and the RTH (in person) groups met in a university building.

The training was designed to be delivered in a standard way across the 2 delivery formats (in-person and VW), including consistent content delivery, supplemental activities, follow-up home activities (eg, review favorite fast-food menu to find healthier choices) and opportunity for peer interaction. For the RTH-VW implementation, participants were provided with an initial technology-focused training session (eg, downloading application, logging in, and use of avatars) and offered a variety of options for follow-up technical support, (eg, videoconferencing, meetings in virtual environment, and telephone support), where needed. Consistent with the RTH delivery, core content sessions in the RTH-VW were generally held in a VW classroom-like setting using the same slide presentations as the RTH to maintain consistency in delivery. Some RTH-VW sessions also included facilitated discussions in immersive locations in the VW to maximize experiential learning (eg, discussing making healthier choices in the fast-food restaurant). The RTH-VW also included opportunities for contextual activities (eg, reading nutrition facts labels in a grocery store), on-demand activities (eg, knowledge check kiosks; activity comparing nutrition facts labels for calories) and on-demand access to educational resources and VW physical activity options (eg, riding a bike, refer to Figure 2).

### COVID-19-Related Modifications

As noted earlier, the COVID-19 pandemic and restrictions resulted in substantial (eg, 9 mo) delay in the study, thereby greatly impacting the implementation (eg, delayed groups, inability to hold in-person sessions, and multiple protocol and IRB revisions). To continue the study during the pandemic and maintain the primary goal of examining the VW implementation, the design was modified to assign participants only to the RTH-VW group and all study activities occurred remotely (ie, videoconference consent process, orientation, postprogram meeting, and training in use of VW application, VW training, and online assessments).

### Assessment Measures and Methods

#### Background and Sociodemographic Information

A baseline survey collected basic background, health-related, and sociodemographic information.

#### Feasibility and Acceptability

Quantitative and qualitative approaches were used to gather participant feedback regarding acceptability and feasibility, especially related to the VW approach. Brief quantitative session feedback surveys tailored for each session’s topics were collected following each session to examine content satisfaction. Participants were asked to rate how well their expectations were met for the session topics with the following response choices: all were met, some were met, very few or none were met. A posttraining survey included quantitative and qualitative questions designed to examine acceptability and feasibility of the training implementation. Example multiple choice questions included: Overall, how would you rate your satisfaction with the information presented during the program? and “Overall, how would you rate the level of interactivity of the program?” with response choices of “poor,” “fair,” “good,” “very good,” and “excellent.” Example open-ended qualitative questions included: “What did you think about the program?”; “What was the most helpful part of the program?”; and “What part of the program needs to be improved?.”

#### Quantitative Outcomes

The primary quantitative pre-post training constructs included training content knowledge and confidence in content and delivery skills. In addition, a secondary exploratory quantitative assessment of behavioral outcomes was conducted. Self-report assessments were conducted electronically throughout the study using Qualtrics [32]software. The pre-post assessments (0, 10‐12 wk) were conducted in-person using a mobile device at a university location before the COVID-19 pandemic and were collected remotely with a survey link during the pandemic. Some measures were omitted due to differences in the measurement approaches (eg, lab-measured BMI vs self-report) and restrictions on daily activities (eg, physical activity measure) due to the evolving COVID-19 pandemic guidance (eg, stay at home, gyms closed, and social distancing).

#### Content Knowledge

Content knowledge was assessed with 22 adapted true or false and multiple-choice knowledge questions taken from the RTH “Toolkit Training Guide” (2008 version) and specific content of the training. Example questions were: “A light bulb size portion equals three servings of vegetables” (true or false) and “In the DPP study, how many minutes of physical activity (for example, brisk walking) did people have to do to prevent or delay type 2 diabetes?” At least 30 minutes every day; 30 minutes 5 days/week; 60 minutes every day; 60 minutes 5 days/week; none of the above (multiple choice). A total correct score was used for analyses (range 0‐22).

#### Confidence in RTH Content and Delivery Skills

This score was computed as an index based on 5 items that asked about the confidence in the training content and implementation of community initiatives and response choices ranged from “not at all confident” to “extremely confident” (range=5‐25). Examples of questions include: “How confident are you that you could present a community awareness event on diabetes prevention?” and “How confident are you in your ability to make content engaging and interactive when delivering a community-based presentation?.”

#### Eating Habits

CHW eating habits were assessed using the Visually-Enhanced Food Behavior Checklist [33] and Rate Your Plate (RYP)-Heart questionnaire [34]. The Visually-Enhanced Food Behavior Checklist aims to “enhance usefulness of food behavior surveys for low-literate populations using visual information processing theories.” It is intended to be used with adults and measures medium-term changes in dietary habits. For the purposes of this study, two variables were examined: (1) daily intake (cups) of fruits and vegetables (ie, composite score of daily fruits and daily vegetable intake in cups; range=none to 6 cups or more) and (2) self-rated eating habits (range=0‐10; poor=0 to excellent=10). The RYP questionnaire includes 24 items (range=24‐72) designed to assess qualitative nutrition information related to the typical food choices, specifically examines intake of 16 food categories, and includes items on food preparation, eating out, and serving sizes (lower score represents healthier eating habits).

#### Stage of Change and Self-Efficacy

Stage of change (SOC) items assessed CHW behavioral motivation (ie, precontemplation, contemplation, and preparation) or achievement of eating habit and physical activity behaviors (ie, action or maintenance stages) across multiple areas, including reducing high fat dairy and protein foods; avoiding sugary beverages; reading Nutrition Facts labels; increasing fruit or vegetables, and getting moderate physical activity. A multi-item index of SOC was used to examine behavioral motivation or change based on coding stage as “Precontemplation=1” to “Maintenance=5”. The SOC items were combined into an overall multi-item SOC index by summing the scores across all 6 SOC items (range=6 to 30).

A multi-item index of behavioral self-efficacy was used to examine confidence in making lifestyle changes (ie, same behavioral areas as SOC). Confidence items had a 5-point response scale, ranging from “not at all confident=1” to “extremely confident=5”. The confidence individual item scores were combined into an overall confidence index by summing the score across all confidence items (range=6 to 30).

#### Adherence and Fidelity

Researchers regularly observed program sessions or activities to monitor fidelity, observe attendance, and examine feasibility aspects of delivering the training. Make-up sessions were offered for each missed session.

#### Posttraining Explanatory Focus Groups

Semistructured focus groups were conducted following the training to gather information on acceptability (eg, likability, tailoring, and suggested refinements), feasibility (eg, facilitators or barriers to participation or VW use), and general feedback. Examples of core structured questions include: (1) What did you think about the VW application? (2) How would you describe this program or VW to a friend or a family member? (3) What was your favorite part and why? (4) What did you think of the different activities in the VW? (5)What did you think of presentations in the VW? And (6) what were your expectations and were they met?

### Analyses

Quantitative and qualitative analysis approaches were used to examine the acceptability, feasibility, and preliminary impact of the VW training model.

#### Quantitative Analyses

The primary focus of the research was learning about the VW approach; however, there were COVID-19-related design or implementation modifications limiting randomization. The descriptive results (eg, mean, SD, sample size, and percentage) are presented for the RTH (in person, n=8) and the combined RTH-VW subsample (n=18). Due to the small sample size, only descriptive statistics are included. For the RTH-VW group, the percent improved and percent unchanged are also described.

#### Explanatory Qualitative Analysis

Posttraining focus group recordings were transcribed and entered into the Dedoose analysis [35] application for coding and analysis. Thematic analysis was used to analyze this data [36]. First, qualitative data was deductively coded across training and implementation-related questions by 2 team members using a priori general categories of positive, negative, and neutral feedback. The total frequency of each global category was calculated. In addition, subthemes were then inductively identified from the positive and negative categories across questions. The coding was reviewed and discussed by 2 team members and inconsistencies were reviewed and resolved.

## Results

### Sample Characteristics

A total of 74 individuals were screened, 25 were ineligible, and 23 eligible individuals did not enroll or were unable to participate. A total of 26 CHWs initiated the study (randomized prepandemic: 8 in-person, 7 VW; during pandemic: 11 VW) and 22 completed the postassessment. The baseline characteristics (N=26 total sample; n=18 combined VW sample) include: all female and African American or Black or African Ancestry; average age of 47 years; education: 3.8% (1/26) high school, 19.2% (5/26) some college, 11.5% (3/26) associate’s degree; 65.4% (17/26) bachelors or advanced degree; and 84.6% (22/26) employed. In addition, 76.9% (20/26) reported daily computer use; 84.6% (22/26) daily internet use; 76.9% (20/26) were very comfortable using a computer; and 80.8% (21/26) were very comfortable using the internet.

### Acceptability and Feasibility Findings

#### Individual Training Session Postsurveys (All Groups)

The findings from the individual postsession surveys combined across all training sessions indicated that greater than 95% of each group (RTH and VW-RTH), on average, reported that their expectations were met across all sessions and content topics. Findings regarding satisfaction with the information provided and level of interactivity of the training sessions across groups indicated that CHWs generally reported satisfication with the information provided (ie, poor or fair=0% [0/22]; good=9.1% [2/22]; very good=40.9% [9/22]; excellent=50% [11/22]) and reported a high level of interactivity of the training (ie, poor=0% [0/22]; fair=4.6% [1/22]; good=18.2% [4/22]; very good=36.4% [8/22]; excellent=40.9% [9/22]). These findings support overall satisfaction with the training experience.

#### Posttraining Survey Questions

Results of the quantitative posttraining acceptability and feasibility survey questions found very positive responses (eg, all respondents rated the general quality of the training as very good-excellent and nearly all (90-100%) liked the number of sessions, length of the sessions, and timing of sessions). All participants agreed or strongly agreed that they would recommend the training to others; and that the training in general and specifically, the healthy eating and physical activity information, was helpful in learning about diabetes prevention to raise awareness in their communities. Participant satisfaction ratings of the information presented during the training were consistent with the other findings (ie, all rated satisfaction as good to excellent).

Exploration of 3 qualitative explanatory survey questions provided additional insights into the acceptability and feasibility of the training. When asked “what did you think about the program?,” the majority of comments were positive with the main subtheme indicating that participants liked the training (eg, “good,” “educational,” and “informative”). Examples of specific feedback on the VW approach included “learning in the VW was enjoyable as you had the opportunity to bond and share in a unique way” and “the online session information presented was good.” Constructive feedback was also offered with the main subtheme focused on improving supplemental resources (eg, bigger font handouts) and one comment suggesting providing more opportunities for avatars to work together in VW activities. When asked about the “most helpful” part of the training, the main subtheme focused on the information provided, especially healthy eating related topics (eg, reading nutrition facts labels) and the 3 core RTH diabetes prevention messages, while other common responses focused on engaging with peers, and positive characteristics of the facilitator. For the question about what “needs to be improved”, the main themes included session scheduling (eg, length and timing) and challenges with using technology (eg, navigating avatar). Regarding barriers experienced in attending the training, the main themes were scheduling challenges or technology challenges.

#### Quantitative Pre-Post Training Outcomes

RTH training-related and exploratory behavioral outcomes are presented in Table 1. Descriptive results are presented for the randomized RTH group and combined RTH-VW group. As can be seen in Table 1, mean pre-post mean values improved for all variables. In addition, for the RTH-VW group, the majority (11/12, 91.7%) improved in knowledge (0/12, 0% unchanged) and half reported increased confidence (2/12, 16.7% unchanged). The majority also experienced improvements across all behavioral outcomes examined.

**Table 1.** Descriptive statistics for pre-post CHW behavioral and training-related outcomes for the in-person training group and the combined VW training groups. Eating habits and fruit and vegetable intake were measured with the Visually-Enhanced Food Behavior Checklist; higher scores represent better outcomes for all outcomes except for the RYP Total; total n for RTH = 8 and total n for RTH-VW =18.

Behavioral and training-related outcomes	Baselinemean (SD, n)	Posttrainingmean (SD, n)	% RTH-VW improvedpre-post	% RTH-VW unchangedpre-post
RTH knowledge (range 0-22)				
RTH^a^	16.13 (2.42, 8)	17.43 (2.23, 7)	—^c^	—
RTH-VW^b^	15.72 (2.76, 18)	18.58 (1.24, 12)	92%	0%
RTH content and delivery confidence (range 5-25)				
RTH	17.38 (3.81, 8)	20.29 (2.50, 7)	—	—
RTH-VW	16.17 (3.85, 18)	18.00 (2.66, 12)	50%	17%
Stage of change index (range 6-30)				
RTH	22.63 (3.81, 8)	24.43 (4.80, 7)	—	—
RTH-VW	20.00 (5.22, 18)	22.75 (3.41, 12)	75%	8%
Self-efficacy index (range 6-30)				
RTH	22.88 (4.19, 8)	26.43 (3.69, 7)	—	—
RTH-VW	20.11 (4.16, 18)	22.42 (3.34, 12)	67%	8%
Eating habits rating (range 0-10)				
RTH	5.63 (2.33, 8)	6.29 (1.70, 7)	—	—
RTH-VW	5.28 (1.65, 18)	6.00 (1.68, 13)	62%	23%
Fruit and vegetable intake (range 0 to 6 cups)				
RTH	1.94 (1.76, 8)	3.00 (1.73, 7)	—	—
RTH-VW	1.06 (0.80, 18)	2.00 (0.91, 13)	69%	31%
Rate your plate total (range 24–72)				
RTH	41.25 (11.02, 8)	40.43 (12.01,7)	—	—
RTH-VW	47.22 (8.75, 18)	40.00 (7.68, 13)	77%	8%

a RTH: Road to Health.

b RTH-VW: Road to Health- virtual world.

c not applicable.

#### Posttraining (Explanatory) Qualitative Findings

The posttraining focus groups included 10 participants from the RTH-VW group. Of the overall positive and negative feedback, 79% (71/90) of the comments were coded positive (19/90, 21% negative). The main positive subthemes focused on the overall experience, interactivity, and content learned, along with the facilitator characteristics. Of the constructive feedback, the main theme was related to navigating the avatar. Table 2 shows the subthemes and sample quotes for each theme.

**Table 2.** Posttraining focus group subthemes and example quotes for each theme. Note: The comments in brackets were added by authors for clarification.

Subthemes	Example quotes
Positive feedback subthemes	
Overall experience	“It was innovative because I know we can’t meet in person, so having class this way I can have my toddlers here playing; I know I can watch them at the same time I am doing something here…I can do two things at once, I don’t have to leave the house”. “Everything. You can go in any time of the night”. [Favorite part] “I actually loved the avatar”.
Interactivity	“Coming together and learning from each other”. “In the evening times, we would just meet up…and go through the things, discuss, and basically well just have fun.” “I like in the classroom when we talked about the meal we had, and how she engaged with everyone.”
Content learned	“I really liked it [fast food restaurant] because, you know, it helped you create this awareness about choosing your options…like to take an opportunity to choose a healthier meal among fast food choices”. “I thought the slide presentations [in virtual world] were pretty good, we are able to follow along with the facilitator”. “Label reading”. [Favorite part]
Constructive feedback subtheme	
Avatar navigation	“In the beginning I believe that it was kind of challenging, I was excited about the virtual world, but navigating around, I think that was my biggest challenge.” “I enjoyed using the application in terms of, you know, moving around and everything…it takes a little getting used to but that was why there was an orientation process at the beginning to help you know and teach you how to move”. “Basically, the biggest problem was learning how to navigate.”

When asked “What were your expectations and were they met?,” all responses were positive. Two example quotes include: “I’ve learned a lot” and “It did in every aspect…I want to help myself because of this program…Now I’m ready to do more for myself.” Examples of quotes about how they would describe the program or VW to a friend or a family member include:

we are represented there…it’s like a village, everything you need is right there…you can go to the grocery store, you can go out in the morning to do some exercise, you can go to the fast-food restaurant to get your food

it is like a classroom…we are not physically present…we all come together in the VW to learn from each other and learn from the doctor [facilitator].

#### Attendance and Fidelity

Observations at sessions confirmed fidelity of content delivery and clarified aspects of feasibility. Nearly all participants of both delivery approaches either attended all sessions or completed make-up sessions.

## Discussion

### Principal Findings

The overall aims of this study were to adapt, implement, and evaluate a VW model to remotely deliver an adapted best-practice CHW training to support CHW’s efforts to raise awareness about diabetes prevention in racial-ethnic minority communities. Integrated quantitative and qualitative findings on the acceptability, feasibility, and preliminary impact of the RTH-VW training model implemented with CHWs shows initial promise of this immersive remote approach.

The findings regarding the acceptability of the training overall were encouraging. The respondents from both delivery approaches (pre-COVID-19 randomized groups) generally provided positive feedback regarding their training experience. For example, strong positive feedback was offered about the general quality, specific content, and interactivity of the training. The postsession feedback for individual sessions for both delivery approaches indicated that nearly all respondents felt their expectations were met across all training topics. VW group participants enjoyed the interactive immersive (eg, label reading in grocery store) aspects of this approach underscoring the unique opportunities available to support learning. Finally, all respondents of the posttraining quantitative survey agreed that they would recommend the training to others. The feedback on acceptability from various sources supports the value of the training overall and both implementation approaches.

While the overall delivery of both training approaches was feasible from a research and implementation perspective (excluding pandemic-related barriers), there remains room for improvement to support participants in being able to fully participate. For example, both groups (prepandemic) continued to experience challenges with attending scheduled sessions related to work and family schedules, possibly contributing to the inconsistency found in the feedback across assessment approaches regarding the length and timing of sessions. This feedback suggests that scheduling is an important consideration in planning any CHW training programs. Suggestions for strategies to remove potential scheduling barriers include allowing each group to identify a mutually convenient time for group members; offering multiple training session options on different days and times, including evenings and weekends; and, as done in this study, offering make-up sessions to support full participation.

Although remote VW delivery may provide unique remote experiential learning opportunities (eg, reading labels in grocery store) and overcome some barriers (eg, transportation, travel time, and parking), constructive feedback was provided on other challenges and areas to further improve feasibility of this virtual approach to maximize interactivity. The explanatory focus group provided a more nuanced understanding of this feedback. For example, some participants experienced challenges with navigating their avatars initially with improvement over time, while a few had continued challenges. Future work should offer more preliminary training in the use of the VW application, as well as continued ongoing support, to enhance feasibility. One useful approach in this study was to meet people, as avatars, in the VW shortly before sessions to review features needed for VW participation (eg, navigating avatar and communicating with other avatars). This required only a few minutes of time and was valuable in helping participants fully engage in the training.

While the results of training-specific outcomes (ie, knowledge, confidence in delivering content and activities) were encouraging, confidence in applying the knowledge could be improved. Future work related to this CHW training and other skill-based trainings might benefit from offering CHWs greater opportunity within the training program to work on tailoring community awareness approaches and activities for their communities, practicing them to enhance confidence, and gathering feedback from other CHWs and facilitators to refine their approaches before community implementation. This may be a useful strategy to support CHWs in applying new content and skills across various CHW training programs.

An exploratory and unique aspect of this study was to include CHWs with similar diabetes risk factors to the community members they serve. The training format (eg, duration, and spacing) was designed to allow participants the opportunity to implement the knowledge and skills learned in the training in their own lives to gain a better understanding of the process and complexity of making healthy lifestyle changes. The exploration of behavior change outcomes showed positive changes in a variety of areas (eg, readiness for change, confidence, fruit, and vegetable intake), thereby underscoring the potential positive impact of the training on the participant’s own lifestyle behaviors. This approach may support CHWs in enhancing empathy and identifying possible behavior change tips for supporting other individuals working on making similar healthy lifestyle changes to reduce the risk of diabetes.

### Strengths and Limitations

A strength of the study was the ability to pivot during the pandemic to continue to deliver the VW approach when in-person restrictions were implemented. This demonstrated that the VW approach was well-suited to implementation during this challenging unanticipated situation and underscored its promise in terms of feasibility and potential to overcome participation barriers. As previously noted, the main limitation in the study was the inability to fully implement the planned randomized design to allow statistical comparison of the 2 approaches (ie, in-person and VW). Another study limitation was the small sample size. The inability to examine all planned outcomes due pandemic-related modifications and influences was also a limitation. Therefore, the results should be interpreted with these caveats in mind.

### Conclusions

Findings on the acceptability, feasibility, and preliminary impact of the RTH-VW training are promising and support continued use of this approach. As noted in the introduction and generally supported in this study, a VW model has many potential advantages in offering trainings for CHWs, such as the potential to be tailored and standardized to disseminate to diverse groups remotely to minimize some common barriers to participation; provides opportunity to deliver engaging interactive contextual or experiential learning activities to help facilitate knowledge, skill, confidence, and empathy; and allows for social interaction and peer-to-peer learning.

Future research will continue to examine this VW training approach incorporating the revised version of the CDC RTH Toolkit resources, additional immersive on-demand activities, constructive feedback from participants, and lessons learned during the implementation experience (eg, greater initial training in using the VW application).

## Abbreviations

3D: three-dimensional
CDC: Centers for Disease Control and Prevention
CHW: community health worker
DDT: Division of Diabetes Translation
ILCHWA: Illinois Community Health Worker Association
RTH: Road to Health
RTH-VW: Road to Health Toolkit- virtual world
RYP: Rate Your Plate
SOC: stage of change
T2DM: type 2 Diabetes Mellitus
UIC: University of Illinois at Chicago
VW: Virtual World
